# Emerging West Nile virus infections in Türkiye

**DOI:** 10.1007/s10096-025-05135-3

**Published:** 2025-04-28

**Authors:** Özlem Alhan, Meliha Meriç Koç, Ayşe Batırel, Emel Yılmaz, Gülden Ersöz, Mehtap Aydın, Merve Üstüner Doğan, Hande Hazır Konya, Üner Kayabaş, Ferit Kuşçu, Zehra Çağla Karakoç, Meryem Şahin Özdemir, Ali Okay Elibol, Esra Kazak, İdil Karaca, Mert Ahmet Kuşkucu, Önder Ergönül

**Affiliations:** 1 Infectious Diseases and Clinical Microbiology, Koç University Hospital, İstanbul, Türkiye; 2 Infectious Diseases and Clinical Microbiology, Başakşehir Çam and Sakura City Hospital, İstanbul, Türkiye; 3 Infectious Diseases and Clinical Microbiology, University of Health Sciences, Kartal Dr. Lütfi Kırdar City Hospital, İstanbul, Türkiye; 4 Infectious Diseases and Clinical Microbiology, Bursa Uludağ University Faculty of Medicine, Bursa, Türkiye; 5 Infectious Diseases and Clinical Microbiology, Mersin University Faculty of Medicine, Mersin, Türkiye; 6 Infectious Diseases and Clinical Microbiology, Ümraniye Training and Research Hospital, Istanbul, Türkiye; 7 Infectious Diseases and Clinical Microbiology, Adana Training and Research Hospital, Adana, Türkiye; 8 Infectious Diseases and Clinical Microbiology, Adnan Menderes University Faculty of Medicine, Aydın, Türkiye; 9 Infectious Diseases and Clinical Microbiology, University of Health Sciences, Kayseri City Hospital, Kayseri, Türkiye; 10 Infectious Diseases and Clinical Microbiology, Çukurova University Faculty of Medicine, Adana, Türkiye; 11 Infectious Diseases and Clinical Microbiology, İstinye University Faculty of Medicine, İstanbul, Türkiye; 12 Medical Microbiology, Koç University Faculty of Medicine, İstanbul, Türkiye; 13 Infectious Diseases and Clinical Microbiology, Koç University Faculty of Medicine, İstanbul, Türkiye

**Keywords:** Acute flaccid paralysis, Encephalitis, Meningoencephalitis, West nile virus infections, Vector-borne disease

## Abstract

**Purpose:**

Türkiye experienced its largest West Nile virus (WNV) infection outbreak in 2024. We described the clinical and laboratory features of human cases with WNV infection collected from eleven tertiary hospitals in Türkiye in 2024.

**Methods:**

The clinical characteristics of the patients were gathered using a structured form in the retrospective study. According to the ECDC case definition of WNV infections, the patients were classified as ‘confirmed’ or ‘probable’ cases. The odds ratio (OR) and 95% confidence interval (CI) for possible mortality predictors in WNV infections were calculated using multivariate logistic regression analysis. *p* < 0.05 was considered statistically significant.

**Results:**

The mean age of the 51 patients was 63.3 ± 13.6 years, and 37 (72.5%) were male. Twenty-six cases (51%) were confirmed, and 49% were probable WNV infection. Forty-eight patients (94.1%) had WNV neuroinvasive disease: 24 (47%) were diagnosed with meningoencephalitis, 20 (39.2%) with encephalitis, one (2%) with meningitis, and seven (13.7%) with acute flaccid paralysis. Twenty patients (39.2%) had movement disorders (tremor, myoclonus, bradykinesia, or rigidity). The case fatality rate was 17.6%. In multivariate analysis, older age (OR: 1.09, CI: 1.03–1.19, *p* = 0.042) and secondary bacterial infection during hospitalization (OR: 10, CI: 1.55–64.95, *p* = 0.015) were associated with fatality.

**Conclusion:**

We highlighted the increasing number of cases and diagnostic challenges by describing the highest number of the patients with WNV infections in Türkiye. Raising awareness among healthcare professionals, facilitating access to diagnostic tests, and developing rapid, reliable, and easily applicable tests would enable early diagnosis and help improve outcomes.

## Introduction

West Nile Virus (WNV), a member of the Japanese encephalitis virus serocomplex group of antigenically related mosquito-borne flaviviruses, is maintained in an enzootic cycle primarily among mosquitoes, particularly *Culex* and *Aedes* species, and birds, with humans and horses as dead-end hosts [1]. The infection is endemic in Africa, Europe, the Middle East, North America, and Western Asia [2].

In Türkiye, 107 WNV infections were reported by the Ministry of Health between 2010 and 2023 [3]. The highest annual number of WNV infections in Türkiye was detected in 2024, and 99 human WNV infection cases were reported to the European Centre for Disease Prevention and Control (ECDC) from the beginning of the year to 20 November 2024 [4]. We retrospectively described the risk factors, patient characteristics, clinical presentations, and case management in the outbreak cases in Türkiye in 2024.

## Methods

We invited infectious disease experts to a face-to-face meeting to present their WNV infection cases at the Koç University İş Bank Infectious Diseases Center (KUISCID) on December 20, 2024. Following this meeting, the clinical and laboratory data of the cases were collected by a structured form from eleven tertiary care hospitals in six provinces of Türkiye (Istanbul, Adana, Aydın, Bursa, Kayseri, and Mersin) until February 13, 2025. Adult patients (≥ 18 years of age) with probable or confirmed WNV infection who were followed up in outpatient clinics or hospitalized in 2024 were included in the study. Patients under the age of 18 years were excluded from the study. Patient data were collected through patient interviews and reviews of inpatient hospital records.

Patient characteristics, comorbidities, presence of immunosuppression, symptoms, and signs at admission, risk factors for WNV infection, length of hospital stay, admission to the intensive care unit (ICU), persistence of symptoms after acute infections, secondary bacterial infections during hospitalization, fatality, and initial laboratory values were collected using the data form. Fatality was defined as a lethal outcome at the end of hospitalization. The patients were discharged with total cure or with some complications and sequelae. The last date for calculating the length of hospital stay and the time of death was February 13, 2025. Healthcare-associated infections developed during hospital stay were defined as secondary bacterial infections. The study was approved by the Koç University Institutional Review Board (reference number: 2025.029.IRB1.005).

The patient samples were sent to the Infectious Diseases and National Arbovirus and Viral Zoonoses Reference Laboratory (Ankara, Türkiye) to diagnose WNV infection. WNV antibodies (IgM, IgG) in serum were studied using the immunofluorescence assay (IFA). The plaque reduction neutralization test (PRNT) was not performed on all samples. The polymerase chain reaction (PCR) was conducted on blood, urine, and cerebrospinal fluid (CSF). The patients were classified as ‘confirmed’ or ‘probable’ cases according to the ECDC case definition of WNV infection [5]. Confirmed cases were defined as a positive PCR for WNV in blood or CSF or a positive PRNT in serum. Probable cases were defined as positive serology (IgM and IgG) for WNV in serum only, negative or unknown PCR in serum and CSF, and negative or unknown PRNT.

Data analysis was performed using the Jamovi Version 2.5 software package. If continuous variables were normally distributed, the mean and standard deviation (SD) were presented; if they were not, the median and minimum-maximum values were presented. Chi-square was used for categorical variables, the T-test was used as a parametric test for continuous variables, and the Mann-Whitney U test was used as a non-parametric test. In the study, the odds ratio (OR) and 95% confidence interval (CI) for possible fatality predictors (older age, comorbidities, presence of immunosuppression, length of hospital stay, admission to ICU, secondary bacterial infections during hospitalization, etc.) in the patients with WNV infection were calculated using univariate logistic regression analysis. *p* values ​​less than 0.05 was considered statistically significant. Variables found to be significant in univariate analysis were included in the logistic regression model for multivariate analysis, and backward selection was performed.

## Results

### Patients’ characteristic

**Fig. 1:**
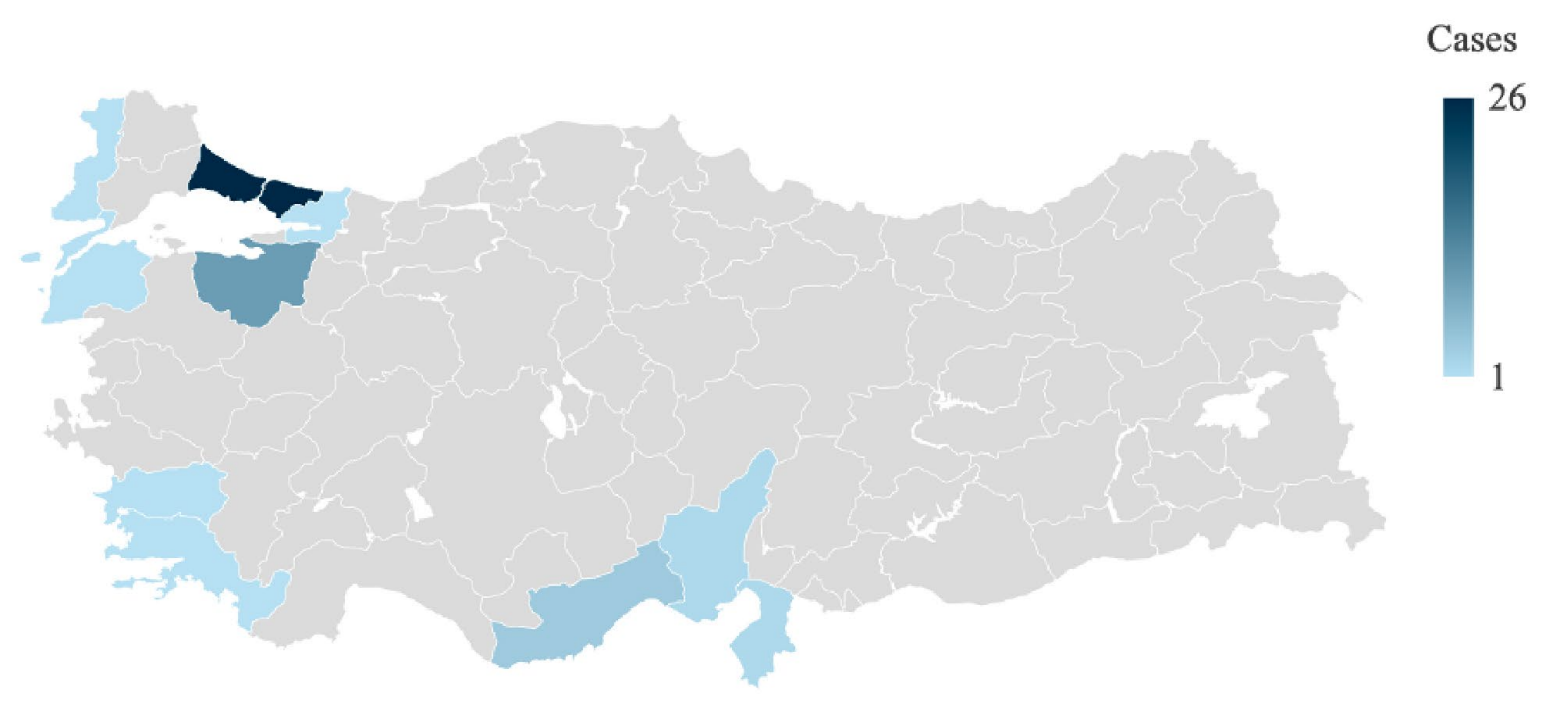
The geographical distribution of 51 WNV cases in Türkiye (Powered by *Bing*. GeoNames, Microsoft, TomTom)

Fifty-one cases from eleven tertiary hospitals were included in the study. The mean age of the patients was 63.3 (SD: 13.6), and 72.5% were male. Except for one patient from Kosovo and one patient from Northern Cyprus, all patients acquired WNV infection locally. Most patients (*n* = 26, 50.9%) lived in İstanbul (Fig. 1). The first patient was detected on July 24, and the last patient on November 5. The most frequent hospital admissions occurred in September (*n* = 25), followed by August (*n* = 13). The median time from symptom onset to hospital admission was 4.5 days (0–22).

Thirty-eight patients (74.5%) had at least one comorbidity. The most common comorbidities were hypertension (49%), diabetes mellitus (35.3%), and cardiac disorders (31.4%). Four patients (7.8%) had malignancies, and one (2%) had solid organ transplantation (Table 1). Seven patients (13.7%) had immunosuppressive drug use (methylprednisolone, azathioprine, or chemotherapy drugs). 51% (*n* = 26) of patients had mosquito bites within the three weeks before symptoms. None of the patients had received a blood product transfusion or organ transplantation in the last 4–8 weeks.

**Table 1:** The characteristics of the patients with West nile virus infections

Patients’ characteristics	*N* = 51 (%)
Age (years), mean (SD)^φ^	63.3 (13.6)
Sex (male)	37 (72.5%)
Time of hospital admission from onset of symptoms (days), median^φ^	4.5 (0–22)
Length of hospital stay (days), median^φ^	12.5 (5-212)
Comorbidities (one or more)	38 (74.5%)
Hypertension	25 (49%)
Diabetes mellitus	18 (35.3%)
Cardiac disease	16 (31.4%)
Renal disease	6 (11.8%)
Neurological disease	5 (9.8%)
Pulmonary disease	5 (9.8%)
Thyroid disease	4 (7.8%)
Liver disease	2 (3.9%)
Immune thrombocytopenic purpura*	2 (3.9%)
Rheumatological disease*	1 (2%)
Malignancy & Transplantation	
Hematological malignancy	2 (3.9%)
Oncological malignancy	2 (3.9%)
Solid organ transplantation	1 (2%)

^φ^Mean was presented with standard deviation (SD), and median was presented with minimum-maximum (min-max)

*Immunosuppressive drugs use (methylprednisolone, azathioprine)

### Clinical features

The most prominent symptoms on admission were fatigue (90.2%), fever (88.2%), and loss of appetite (64.7%). 52.9% of patients (*n* = 27) had weakness in the extremities. Forty-one patients (80.4%) had confusion or loss of consciousness, and 14 patients (28.6%) had nuchal rigidity on admission (Table 2).

**Table 2:** The symptoms and signs of the patients with West nile virus infections

Patients’ symptoms and signs	*N* = 51 (%)
Fatigue	46 (90.2%)
Fever (≥ 38 °C)	45 (88.2%)
Confusion or loss of consciousness	41 (80.4%)
Loss of appetite	33 (64.7%)
Headache	30 (58.8%)
Nausea and vomiting	28 (54.9%)
Weakness in the extremities	27 (52.9%)
Myalgia	14 (27.5%)
Nuchal rigidity	14 (28.6%)
Abdominal pain	10 (19.6%)
Syncope	9 (17.6%)
Diarrhea	9 (17.6%)
Weight loss	8 (15.7%)
Arthralgia	7 (13.7%)
Back pain	6 (11.8%)
Pharyngitis	6 (11.8%)
Constipation	5 (9.8%)
Lymphadenomegaly	2 (3.9%)
Rash	2 (3.9%)

Most patients (*n* = 48, 94.1%) were diagnosed with West Nile Neuroinvasive Disease (WNND). Twenty-four patients (47%) had meningoencephalitis, 20 patients (39.2%) had encephalitis, one patient (2%) had meningitis, and seven patients (13.7%) had acute flaccid paralysis. Four patients (7.8%) had cranial nerve lesions involving the second, third, fifth, and sixth cranial nerves. Twenty patients (39.2%) had movement disorders; seven patients (13.7%) had hyperkinetic symptoms (tremor, myoclonus, or hyperactivity); eight patients (15.7%) had hypokinetic symptoms (rigidity, bradykinesia), and five patients (9.8%) had mixed features. Ten patients (19.6%) had ocular involvement (retinitis, involvement of cranial nerves II, III, and VI, conjunctivitis, or occlusive vasculitis).

### Laboratory, neuroimaging, and neurophysiological evaluation

Thirty patients (60%) had leukocytosis. The median level of leukocytes was 11.2 × 1000/µL (1.28–92.2). The median CRP was 11.1 mg/L (0.2–321), and the median procalcitonin level was 0.1ng/mL (0.02–1.78). CSF studies were performed in 43 cases. Of the 43 CSF samples, 20 were clear, 16 were cloudy, and four were hemorrhagic (three were unknown). 95.1% of samples had an elevated protein level (≥45 mg/dL); the median CSF protein level was 114 mg/dL (17–692). The median CSF glucose level was 60.5 mg/dL (18–153). Four patients (11.1%) had CSF glucose levels ≤ 40% of concurrent blood glucose. CSF leukocyte counts ranged from 10 to 3400/mm³ and were usually lymphocyte predominant (77.4%).

Most patients (*n* = 48, 94.1%) underwent brain magnetic resonance imaging (MRI). Of these, 45.8% (*n* = 22) had unremarkable MRI findings other than cerebral atrophy. Six patients with meningoencephalitis had leptomeningeal enhancement, and one had ventriculitis on MRI. Sixteen patients had areas of chronic ischemic or nonspecific gliotic hyperintensity in the cerebral white matter, and two had microhemorrhages on MRI. Three patients had acute ischemic diffusion restrictions in the thalamus, periventricular areas, and centrum semiovale on diffusion-weighted imaging.

Sixteen patients underwent an electroencephalogram (EEG). In 43.7% of them, EEG examination revealed findings within physiological limits. Nine patients had nonspecific abnormalities (generalized disorganization or paroxysmal abnormalities) on EEG.

### West nile virus diagnostic tests

We could send WNV antibody in serum from 51 patients, WNV PCR in serum from 48 patients, and WNV PCR in urine from 29 patients. CSF samples were taken from 43 patients, but WNV PCR in CSF from 21 patients was sent to the reference laboratory. The CSF sample of a patient living in Kosovo was not examined in the reference laboratory in Türkiye. WNV microneutralization tests were performed on 16 patients. Diagnostic tests for WNV infection were requested a median of 10 days (2–40) after symptom onset. PCR and serology results of the patients were summarized in Table 3. Two patients had negative WNV IgM; one of them also had negative WNV IgG in serum, but both had positive WNV PCR in CSF samples. Twenty-six cases (51%) were confirmed WNV infection, and 25 were probable cases.

**Table 3:** The results of serology and polymerase chain reaction tests of 51 patients with West nile virus infections

Tests	Positive tests / Studied tests
WNV serum IgM, IFA	49 / 51
WNV serum IgG, IFA	50 / 51
WNV plasma/serum PCR	7 / 48
WNV urine PCR	18 / 29
WNV CSF PCR	4 / 21
PRNT	16 / 16

Cerebrospinal fluid: CSF, Indirect fluorescent antibody: IFA, Plaque reduction neutralization test: PRNT, Polymerase chain reaction: PCR, West Nile Virus: WNV

### Disease course and outcomes

Out of 51 patients, 48 (94.1%) were hospitalized. The median length of hospital stay was 12.5 days (5-212). Eighteen patients (35.3%) were admitted to the ICU, and fourteen patients (27.4%) required invasive mechanical ventilation. The case fatality rate (CFR) was 17.6% (*n* = 9). All deceased cases were over 60 years of age and had at least one comorbidity. The mean time from hospital admission to fatality was 43.1 days (SD:31.3). In univariate logistic regression analysis, fatality was associated with older age (OR:1.1, CI: 1.01–1.19, *p* = 0.02), diabetes mellitus (OR:5, CI: 1.07–23.3, *p* = 0.04), pulmonary disease (OR: 10, CI: 1.37-72, *p* = 0.023), cardiac disease (OR:6.4, CI: 1.34–30.3, *p* = 0.019), admission to ICU (OR:25.6, CI 2.8–230, p:0.004) and other secondary bacterial infections during hospitalization (OR:10.15; CI 1.8–57.1, *p* = 0.009). In multivariate analysis that was performed by backward selection, older age (OR:1.09, CI: 1.03–1.19, *p* = 0.042), and secondary bacterial infections (OR:10, CI: 1.55–64.95, *p* = 0.015) were found to be associated with the fatality.

Seventeen patients (35.4%) had additional infections during hospitalization, including ventilator-associated pneumonia, cholecystitis, catheter-related bloodstream infection, preseptal cellulitis, thyroiditis, bacterial meningitis, and reactivation of latent TB. Twenty-two patients (51.2%) in the survivor group had sequelae after WNV infection, such as balance disorder, slow gait, vision or hearing loss, incontinence, and coma. Fourteen patients (32.5%) in the survivor group had persistent symptoms after WNV infection, including memory impairment, headache, fatigue, depression, and loss of appetite, within three months of follow-up.

## Discussion

The highest annual number of WNV infection cases in Türkiye was reported in 2024 [4]. We described the clinical and laboratory features of 55 patients with WNV infection. In our study, the mean age of patients was 63.3 years, and 94.1% of the patients were diagnosed with WNND. The CFR was 17.6%, and all deceased cases were over 60 years of age and had at least one comorbidity. In the multivariate logistic regression analysis, older age and secondary bacterial infections during hospitalization were found to be associated with fatality. There are areas for improvement in WNV infection in Türkiye; firstly, the awareness of the infection should be increased. Secondly, diagnostic capacity should be enhanced, especially rapid and easy-to-access tests are needed.

WNV infection has been notifiable at the European Union (EU) level since 2008, and EU countries report human cases to the ECDC [6]. According to ECDC reports, the highest number of WNF infections in the EU and EU enlargement countries was reported in 2018, followed by 2024 and 2022 [4]. Between 2010 and 2018, 3,849 patients were detected in the EU and EU enlargement countries; 78% of them were confirmed cases. The median age of WNV cases was 66 years, with a male-to-female ratio of 1.5. Most infections were detected from early summer to early autumn, reaching a clear peak in August [7]. In our study, the median age of the cases was 63.3 years, and the male-to-female ratio was 2.6. Most cases were detected in September, followed by August.

WNV causes asymptomatic infection in approximately 75% of patients, West Nile fever in 25%, and WNND occurs in less than 1% of patients [8, 9]. In our study, 94.1% of the patients had WNND. Asymptomatic or mild cases are thought to have remained undetected because they didn’t seek healthcare or were misdiagnosed due to non-specific symptoms. On the other hand, the high rate of WNND among the cases suggests that WNF cases were much more than detected. Risk factors for WNND include older age, male gender, immunosuppressive conditions, and chronic diseases [10]. Danis et al. reported that the risk of developing WNND was 50-fold higher in the elderly (over 80 years) than in the younger age group (under 20 years) [11]. In our study, the median age of patients with WNND was 66 years (24–87), 75% had at least one comorbidity, and 14.6% used immunosuppressive drugs. According to European Surveillance System data, in 2,916 WNND cases between 2006 and 2021, CFR was detected as 13.1% and 18.4% among overall and hospitalized cases, respectively [12]. Older age, male gender, hospitalization, and living in Türkiye, North Macedonia, Czechia, Kosovo, and Bulgaria were found to be associated with death in patients with WNND [12, 13]. In our study, older age and secondary bacterial infections during hospitalization were found to be associated with fatality.

The patients with WNND frequently have movement disorders, which can be hyperkinetic (e.g., tremor, myoclonus) or hypokinetic (Parkinsonian bradykinesia, rigidity). In our study, 39.2% of patients with WNND had movement disorders: 13.7% had hyperkinetic disorders,15.7% had hypokinetic disorders, and 9.8% had mixed features. WNV infections may present with certain ocular findings, including chorioretinitis, uveitis, retinal hemorrhages, vitritis, and occlusive retinal vasculitis [14]. In our study, 19.6% of patients with WNND had ocular findings. WNV infections cause long-term physical, cognitive, and functional sequelae. The most common sequelae reported were muscle weakness, fatigue, myalgia, headache, balance problems, memory loss, depression, difficulty concentrating, and difficulty performing activities of daily living [15]. In our study, 51.2% of the survivor group had sequelae, and 32.5% of them had persistent symptoms within three months after WNV infection.

The first cases of WNV infection in Türkiye were reported in Manisa in 2010 [16]. According to Ministry of Health data, cases peaked twice between 2010 and 2023, in 2010 and 2018 [3]. Two case series of WNV infections have been reported in Türkiye so far. Between July and November 2010, 47 cases of WNV infection (35 probable cases, 12 confirmed cases) were detected in 15 provinces in Türkiye. The median age of the patients was 58 years; 36.1% of the patients were over 70 years of age, and 68% were male. Most patients (85.1%) were diagnosed with WNND, and the CFR was 21% [16]. In a study of 17 cases of WNV infection (4 confirmed cases, 13 probable cases) between 2017 and 2019, 82.3% of patients had comorbidities. The most common symptoms were fever (88.2%) and headache (66.7%). All patients had neuroinvasive disease (14 with meningoencephalitis, 2 with meningitis, 1 with flaccid paralysis), and mortality was 11.7% [17].

Although the first case was reported in 2010, many serological studies conducted before the human case indicate that the infection has been present in Türkiye for a long time. In 1964, the mean positivity of the hemagglutination inhibition test against WNV in three provinces (İzmir, Diyarbakır, Adana) was 35.6% [18]. In 1973, WNV antibodies were detected in 41.8% of 763 samples from the Southeastern Anatolia Region using the hemagglutinin inhibition test [19]. These studies may not reflect real numbers for WNV seropositivity because the samples weren’t confirmed by PRNT. As a result of PRNT analysis of samples taken from 179 patients who applied to the outpatient clinic in the Southeastern Anatolia Region in 2005, WNV seroprevalence was found to be 9.5% [20]. In another serologic survey conducted on the Syrian border of Türkiye in 2009, microneutralization test positivity was found to be 17% [21]. In the same year, a study investigating WNV seroprevalence in blood donors in Central Anatolia found a seropositivity of 0.8% with the PRNT test [22]. In a population-based study conducted in Manisa in 2014, WNV IgG antibody positivity was detected in 3.8% [23].

Ecological studies are critical to the control of WNV infection. WNV circulates among birds and bird-feeding mosquitoes, commonly *Culex pipiens*. Human exposure to infected mosquitoes depends on a variety of factors, including mosquito features (larval developmental time, adult lifespan), mosquito abundance, weather, and climate features that affect vector survival, annual variations in WNV transmission cycles among avian reservoirs, land use change, lower socioeconomic status, occupation, and climate change [24]. The unusually warm summer in Europe in 2018 is thought to have caused the WNV transmission season to last longer than in previous years and to have caused a peak in the number of cases [7, 25]. Climate change affects the transmission of mosquito-borne diseases by extending the transmission seasons, reducing the abundance of mosquito predators, and altering the development of vectors and pathogens, mosquito habitats, and geographic ranges [26]. In a study conducted on mosquito sampling in Edirne, located in the northwestern part of Türkiye, the WNV infection rate in *Cx. pipiens* pools was found to be 36.3% in 2012 [27]. *Aedes albopictus*, which also transmits WNV at certain temperatures, is expanding its distribution area in Türkiye every year and is expected to further expand its distribution throughout Türkiye in the next few years [28]. In the arboviral screening study in northeastern Türkiye, 89.6% of the collected mosquitoes were *Aedes albopictus*, and WNV infection was detected in 7.7% of the mosquitoes [29]. WNV outbreaks among equids and birds are reported to ECDC from the EA/EEA countries, but not from Türkiye. Surveillance of mosquitoes, birds, and equids would be useful to provide better insight into WNV ecology and circulation in Türkiye.

There are several challenges in the diagnosis of WNV infection. Firstly, less than 1% of patients with WNV infection have neuroinvasive diseases, and other patients are asymptomatic or have mild symptoms. No specific symptoms or signs can help differentiate other etiologies of meningoencephalitis in patients with WNV. In countries where West Nile Virus has become endemic in recent years, such as Türkiye, it should be considered in cases of meningitis and encephalitis between March and November. The second challenge associated with diagnosis is the accessibility of WNV tests. Serological and molecular testing for WNV infection is conducted exclusively at a reference laboratory in Ankara, the capital of Türkiye. Since performing diagnostic laboratory tests in a single center delays diagnosis, regional testing capacity needs to be enhanced. The third challenge is to confirm the diagnosis of WNV infection. The limitation in the number of confirmed cases is attributable to the fact that PRNT is not conducted on every sample, coupled with the challenges associated with detecting positivity in PCR tests, which arise from the short duration of viremia in humans.

In this national study, we included more than half of the cases that were reported to the ECDC. The strongest feature of our study was the inclusion of detailed clinical characteristics of the patients. Having a retrospective design was a limitation. For instance, we couldn’t record all the features uniformly, and a few patients lacked laboratory results.

## Conclusions

The highest number of WNV infections in Türkiye was reported in 2024, and we presented the features of 51 cases with a CFR of 17.6%. Interdisciplinary studies are needed, including surveillance of mosquitoes, birds, equines, and human cases. Training of healthcare workers is necessary for the detection and management of cases. Facilitating access to tests and developing new diagnostic tests that are rapid, reliable, and easy to apply are important issues that need to be prioritized. Increasing test availability would lead to more confirmed cases, better epidemiological measures, earlier diagnosis, optimal clinical care, and improved outcomes.

## Data Availability

No datasets were generated or analysed during the current study.
